# *In vitro* and *ex vivo* evaluation of the biological performance of sclerosing foams

**DOI:** 10.1038/s41598-019-46262-0

**Published:** 2019-07-08

**Authors:** Elisabetta Bottaro, Jemma A. J. Paterson, Luciano Quercia, Xunli Zhang, Martyn Hill, Venisha A. Patel, Stephen A. Jones, Andrew L. Lewis, Timothy M. Millar, Dario Carugo

**Affiliations:** 1 Faculty of Engineering and Physical Sciences, University of Southampton, Southampton, UK; 2 Faculty of Medicine, University of Southampton, Southampton, UK; 3 Computer Science Department, University of Bari, Bari, Italy; 4 Institue for Life Sciences (IfLS), University of Southampton, Southampton, UK; 5 Biocompatibles UK Ltd., Lakeview, Riverside Way, Watchmoor Park, Camberley, UK

**Keywords:** High-throughput screening, Peripheral vascular disease, Biomedical engineering

## Abstract

Since the first reports on foam sclerotherapy, multiple studies have been conducted to determine the physical properties and behavior of foams, but relatively little is known about their biological effects on the endothelial cells lining the vessel wall. Moreover, a systematic comparison of the biological performance of foams produced with different methods has not been carried out yet. Herein, a 2D *in vitro* method was developed to compare efficacy of commercially available polidocanol injectable foam (PEM, Varithena) and physician-compounded foams (PCFs). Endothelial cell attachment upon treatment with foam was quantified as an indicator of therapeutic efficacy, and was correlated with foam physical characteristics and administration conditions. An *ex vivo* method was also developed to establish the disruption and permeabilisation of the endothelium caused by sclerosing agents. It relied on the quantitation of extravasated bovine serum albumin conjugated to Evans Blue, as an indicator of endothelial permeability. In our series of comparisons, PEM presented a greater overall efficacy compared to PCFs, across the different biological models, which was attributed to its drainage dynamics and gas formulation. This is consistent with earlier studies that indicated superior physical cohesiveness of PEM compared to PCFs.

## Introduction

Chronic venous insufficiency (CVI) is the global term to describe failure of venous drainage. CVI can occur in the superficial venous system, the deep venous system (veins within the muscle compartment of the leg), or both. Superficial venous incompetence of the leg may involve any of the veins of the superficial venous system, which includes the great saphenous vein (GSV), small saphenous vein (SSV), and their tributaries. The outward manifestation of superficial venous incompetence is often referred to as varicose veins^1,2^. Sclerotherapy has been employed (along with surgery, radiofrequency and laser ablation) to treat all types and sizes of varicosities by damaging the endothelial lining of the vein wall, causing shrinkage of the treated vessel and leading to the development of new veins.

Sclerosing agents in the form of liquid surfactant solutions have been largely used in the clinic^3^. Since the first reports of the ability to create stable foams from detergent-type sclerosants, foam sclerotherapy has however become widely adopted by clinicians, largely replacing the traditional injection of liquid sclerosants^4–6^. This change in clinical practice is due to several advantages of foamed sclerosing agents when compared to their liquid counterparts^7^. When a liquid sclerosant is injected into a vein, it is rapidly diluted by the circulating blood volume. It has been demonstrated that the interaction with blood decreases the efficacy of sclerosants, due to binding with plasma proteins that ultimately reduces the number of active molecules^8–11^. A foamed sclerosant on the other hand, is able to displace blood rather than mixing with it, increasing the contact time of a higher concentration of active agent with the vein wall and thus resulting in greater efficacy. For these reasons, in foam sclerotherapy, lower concentrations of sclerosant are required to obtain the same therapeutic effect as in their liquid counterpart, reducing the prevalence of side effects associated with higher concentrations^12^.

Over the last 60 years, different foam production methods have been proposed. The two most common techniques that clinicians employ to generate physician-compounded foams (PCFs), are the double syringe system (DSS) and the Tessari method (TSS)^13^. DSS involves passing the sclerosant liquid and a gas between two syringes joined by a straight connector, whereas in the Tessari method the connector is replaced with a three-way valve. Recently, automated production methods have been introduced, such as polidocanol injectable foam (PEM) (Varithena, Provensis Ltd, a BTG International group company), which is designed with a foam generating device for producing a 1% polidocanol O_2_:CO_2_ (65:35) based foam (1:7 liquid:gas ratio), which is virtually nitrogen-free (<0.8%).

The most clinically employed sclerosants are liquid polidocanol (POL) and sodium tetradecyl sulfate (STS) at concentrations of 0.5% to 3% by volume. PCFs are typically produced with carbon dioxide (CO_2_) or room air (RA) at different liquid:gas volume ratios (1:4, 1:3 and 1:7) by phlebologists^13^. CO_2_ foam presents a shorter half-life compared to RA foam^14^, but the latter is associated with higher incidence of side effects including visual disturbances, chest tightness, cough, and dizziness^15^. In addition, RA foam has a high nitrogen content (>70%), which increases the risk of microembolism because of greater bubble persistence due to the low solubility of nitrogen in blood^16^.

The ideal sclerosing foam should offer desirable physical and biological performance. From a physical perspective, it should be sufficiently cohesive to completely fill the vein lumen upon injection, acting as a piston to displace blood rather than mixing with it^17^. Moreover, it should be sufficiently stable to maintain maximal activity from preparation to administration, but short-lived enough to cause limited side effects^18^. Previous studies have shown that these properties strongly depend on the foam manufacturing method, the gas formulation, the gas-to-liquid volume ratio, and the type and concentration of surfactant^14^. From a biological perspective, the ideal foam should damage all endothelial cells in the treated area, with negligible off-target and systemic effects^19^. Greater endothelial damage is preferable as the smooth muscle layer of the vein wall can theoretically regenerate a partially compromised endothelium, and endothelial cells can migrate long distances to re-establish a functional conduit^19^.

It has been previously postulated that biological effects of sclerosing foams may depend on their physical characteristics^20,21^. However, whilst numerous studies have been conducted to determine the physical and mechanical properties of foams (i.e., foam dwell time, drainage time, bubble size distribution, etc.)^17,20,22,23^, relatively little is known about their biological effects on the endothelial cells lining the vessel wall. It is widely accepted that sclerosants disrupt the cell membrane causing (i) endothelial cell (EC) death microscopically, and (ii) macroscopic vein wall damage, such as disruption of the subintima (i.e. the elastic tissue located underneath the endothelium) and mild alterations of the smooth muscle layer^24,25^.

Limited *in vitro* studies have been performed to investigate the microscopic effects of sclerosants^11,26–28^. Most of these studies involve culturing of ECs over a plate, exposing cells to sclerosants, followed by staining with dyes to evaluate cell membrane lysis or cell death (see Table 1).

**Table 1:** Summary of *in vitro* studies performed to investigate the microscopic effects of sclerosants.

Author	Kobayashi^27^	Mol^28^	Parsi^11^
Cell type	BAECs Bovine aortic endothelial cells	HUVECs Human umbilical vein endothelial cells	HMEC-1 Human microvascular endothelial cell line
Treatment	Liquid 3% POL or 1% STS (and further dilutions)	Liquid POL (1.5%, and further dilutions)	Liquid STS (3%) and POL (3%, and further dilutions)
Treatment Time	0–1 hr	5 s	15 min
Method of Administration	Injection	Injection	Injection
Analysis/Outcome	Fluorescent dye measurement/cell death	Dye measurement /cell death	Dye measurement/cell lysis
Quantification method	Fluo4/AM and DAF-FM/DAPI	MTT/Trypan blue/DiI/ICAM	Leishman’s stain

Kobayashi *et al*. determined an inverse correlation between sclerosant concentration and the minimum contact time required to cause endothelial cell death^27^. They found that upon exposure to 1.5% POL liquid solution, cell death occurred after 15 seconds, while a 0.3% POL solution required 15 minutes to achieve the same effect. At very low concentrations of POL (0.003%) cell death did not occur, even after 1 hour of exposure. In a similar study by Mol *et al*.^28^ it was found that almost all cells died after 5 seconds of exposure to 0.025% POL, whereas at lower concentrations (<0.0125%) cell death occurred within 2 minutes. Both studies demonstrated that treatment time is dependent on POL concentration, although there were some significant differences in the time required to cause endothelial cell death *in vitro*.

Parsi *et al*. investigated the deactivating effect of circulating blood cells on the lytic activity of detergent sclerosants^11^. ECs were exposed for 15 minutes to different mixtures of sclerosants with blood, and subsequently labelled with a Leishman’s stain. Results showed that the number of non-lysed cells was concentration-dependent, and that POL had a lower lytic action compared to STS.

Notably, these earlier *in vitro* studies only focused on liquid sclerosants; thus, a systematic comparison of the biological effects induced by foamed sclerosants has not been performed yet.

With respect to the macroscopic effects of sclerosants, several histological studies have been reported, demonstrating that POL and STS significantly compromise the vein wall’s integrity by damaging the endothelium^25,29–31^. In most studies, segments of vein were treated with sclerosant, and stained afterwards with dyes to evaluate damage to the vessel wall (see Table 2).

**Table 2:** Summary of histological studies performed to investigate the macroscopic effects of sclerosants.

Author	Orsini^31^	Ikponmwosa^30^	Erkin^29^	Whitely^25^
Part treated	Vein segment	Vein segment	Vein segment	Vein segment
Treatment	3% STS foam (TSS 1:4)	1% and 3% STS foam	0.1–3% POL foam (TSS)	0.5–3% liquid STS and POL
Treatment Time	2–15–30 min	5 min	5 min	1–10 min
Method of Administration	Filling the vein	Injection with cannula	Soaking	Filling the vein
Analysis/Outcome	Histological/Staining/Wall damage	Histological/Wall damage	Histological/Wall damage	Histological/Staining/Wall damage
Quantification method	H&E (Hematoxylin and eosin stain) and with Weigert and Weigert-Van Gieson histochemical methods	H&E (Hematoxylin and eosin stain)	H&E (Hematoxylin and eosin stain)	Up-regulation of p53 and intracellular adhesion molecule-1 (ICAM-1)

Orsini and Brotto have analyzed the immediate effects on the saphenous vein wall *in vivo*, upon sclerotherapy with STS foam produced with TSS at 1:4 liquid:RA ratio^31^. Vein wall damage was rapid, with complete disruption of the endothelium occurring within the first 2 minutes. In the successive 15 and 30 minutes, edema of the subintima was observed, accompanied by progressive separation from the tunica media and initial formation of a thrombus.

Ikponmwosa *et al*. treated vein segments with 1% or 3% STS foam produced using TSS, at a 1:3 liquid:RA volume ratio^30^. Upon exposure to STS foam for 5 min, the percentage of EC loss was 86.3% (1% STS) and 92.2% (3% STS), whilst the percentage of tunica media injury was 8.9% (1% STS) and 12% (3% STS).

Erkin *et al*. treated varicose vein segments with a selected concentration of POL foam produced with the TSS method, at 1:4 liquid:RA ratio. Vein segments were immersed in foam for 5 minutes, and subsequently examined^29^. Treatment with POL foam caused endothelial swelling, necrosis, and intimal thickening. However, these effects were not statistically correlated to the concentration of sclerosant, except for the presence and extent of necrosis.

Whiteley *et al*. treated *ex vivo* human varicose veins with 1% or 3% STS and POL, for 1 or 10 minutes^25^. Cell death and medial damage were directly correlated to surfactant concentration and treatment time. POL caused less damage to the endothelium and smooth muscle cells compared to STS.

Overall, these histological studies demonstrated the qualitative effects of the interaction between sclerosing agents and the vessel wall. Quantitative analyses mostly relied on microscopic measurements, which were however limited to regions of interest within the treated vessel. As for the *in vitro* studies, therapeutic effects were largely dependent on treatment time and sclerosant concentration, although treatment timescales differed between investigations. This could be due to differences in the physical properties of the sclerosing agent used and the experimental conditions. To the best of the authors’ knowledge, there is no comparative quantitative analysis between different foam production or administration methods, or attempt to correlate physical with biological performance of sclerosing foams. This is also reflected in the lack of clinical studies comparing efficacy and safety of different foam production methods.

Herein, we propose two methods for quantifying sclerosant-induced disruption of the endothelial layer *in vitro* and *ex vivo*. Using the *in vitro* model, the therapeutic efficacy of different polidocanol-based sclerosing agents was investigated, and correlated with their physical characteristics and administration protocols. Therapeutic efficacy was subsequently evaluated within a more complex *ex vivo* model. For the first time, a comparison between different foam production techniques has been performed, by employing biological models with different levels of complexity. Results from this study can provide clinicians with some fundamental understanding of how different foam formulations may perform in the body.

## Results

### *In vitro* evaluation of the biological performance of sclerosing agents

In the first step of the study, a method replicating the clinical treatment procedure was designed in order to investigate the biological effects of sclerosants on a two-dimensional (2D) endothelial model (see Methods section for additional details). The mechanism of action of sclerosing agents relies on endothelial damage; therefore, endothelial cell attachment was employed as a metrics for therapeutic efficacy. Since detached endothelial cells are known to undergo apoptosis, cell attachment was considered as an indicator of cell viability^19^. Therefore, a lower percentage of attached cells upon treatment indicated a more effective sclerosing agent.

Firstly, the repeatability of the method was assessed by fixing the injection and treatment parameters (PEM foam, 15 seconds of treatment time, and 1 mL of foam injected without needle) and repeating the experiment six times. Results showed consistency of foam performance across multiple independent repeats (see Fig. S1(A)).

**Figure 1:**
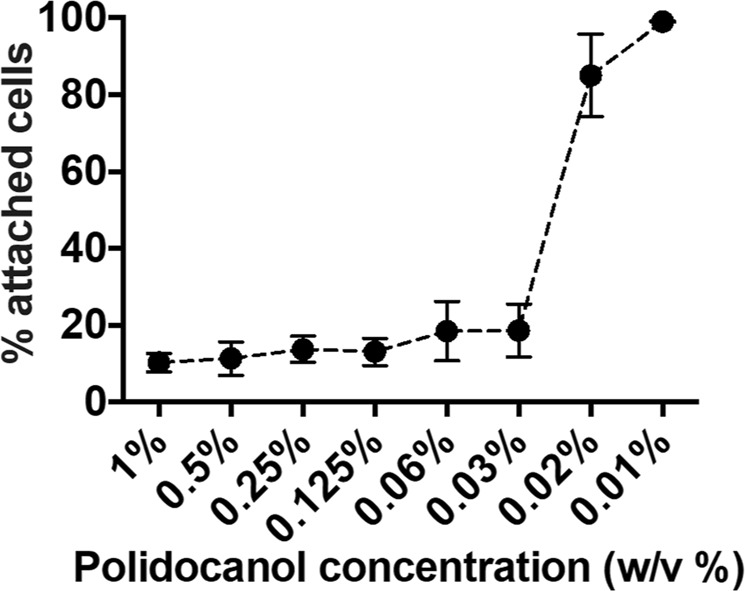
*In vitro* evaluation of the effect of liquid polidocanol concentration on HUVECs. 1% polidocanol (in PBS) was serially diluted seven times using PBS. HUVECs were treated with 1 mL polidocanol solutions for 15 seconds. Data are reported as percentage of attached cells (compared to untreated cells), determined via methylene blue method. The experiment was repeated six times, and results are reported as mean value ± standard deviation.

Subsequently, the sclerosing efficacy of liquid POL was investigated. A 1% POL solution was serially diluted in PBS in order to identify the minimum effective and 50% inhibitory concentrations (15 seconds treatment duration, and 1 mL of sclerosant injected without needle). Figure 1 shows that POL 1% is still effective even after five serial dilutions (0.03% final volumetric concentration), removing >50% of cells in a well. Concentrations of foam below 0.02% rendered the treatment ineffective (85 ± 10% of attached cells). A 50% inhibitory concentration of 0.024% was determined from these experiments.

**Figure 2:**
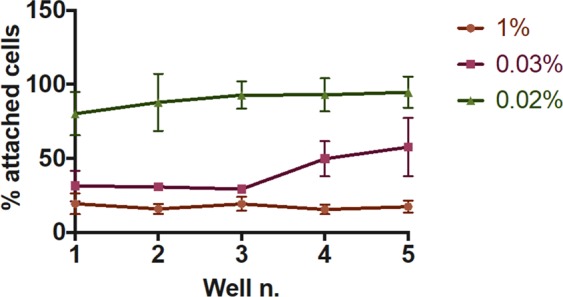
Assessment of polidocanol depletion *in vitro*. POL solutions at different volumetric concentrations (1%, 0.03% and 0.02%, in PBS) were injected into one well and left for 15 s to interact with HUVECs. They were then removed and injected in a neighbouring well. The process was repeated to treat five wells serially, in order to investigate potential depletion of active polidocanol. Data are reported as % of attached cells, determined via methylene blue assay. The experiment was repeated six times, and results are reported as mean value ± standard deviation.

An additional experiment was designed to investigate the extent of polidocanol ‘depletion’, potentially due to the interaction with cell medium constituents or intercalation within cell membrane fragments. In these experiments, 1 mL of liquid POL was injected into one well and left for 15 seconds. The solution was then transferred into a neighbouring well, and the process was repeated in order to treat five wells in series. As shown in Fig. 2, the 1% polidocanol solution maintained the same efficacy after five serial injections (only 17.5 ± 4.0% of cells remained attached after the 5^th^ injection). The experiment was repeated using a lower POL concentration of 0.03%. Results demonstrated that depletion of active POL occurred, as the percentage of attached cells after treatment increased from 29.3 ± 2.0% (3^rd^ injection) to 49.82 ± 11.8% (4^th^ injection) and 57.7 ± 19.6% (5^th^ injection). Reducing the POL concentration further (to 0.02%) resulted in a similar trend, although the change in percentage of attached cells was less significant because of the reduced effectiveness of the sclerosing solution (coherently with the results shown in Fig. 1).

**Figure 3:**
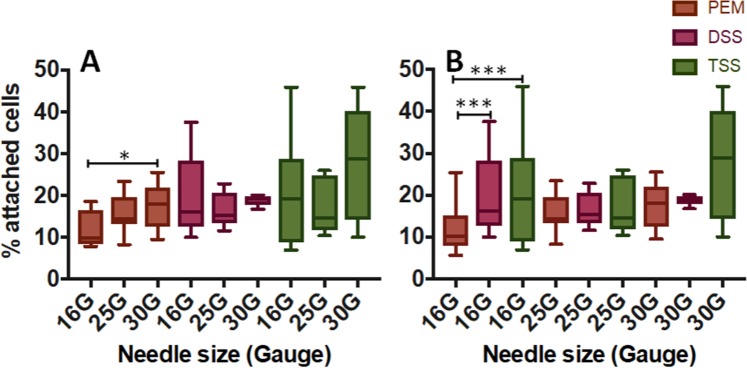
*In vitro* evaluation of the effect of needle bore size on HUVECs, using different types of foam. Treatment efficacy was evaluated at varying injection needle diameters (30G, 25G and 16G) and foam production methods [PEM (brown), DSS (pink), and TSS (green)]. Experiments were performed with a 15 seconds exposure time and 1 mL of injected foam. Data are reported (Tukey’s box plot) as % of cells attached after treatment (compared to untreated cells), determined via methylene blue method. The effect of needle bore size (for each foam production method) is illustrated in (**A**), while a comparison between foam production methods (for each needle bore size) is illustrated in (**B**). The experiment was repeated six times. One asterisk (*) indicates p ≤ 0.05, three asterisks (***) indicate p ≤ 0.001.

The usage of injection needles with different bore size was also investigated, because of their potential effect on foam size and stability. Cells were exposed to 1 mL of PEM foam for 15 seconds, either with or without a needle. Firstly, a needle with the greatest bore size in the range investigated was employed (16G). Fig. S2 shows that the presence of a 16G needle had a negative impact on foam treatment efficacy (i.e., the percentage of attached cells upon treatment increased from 10.12 ± 2.2% to 16.26 ± 3.0%; p < 0.001). Therefore, in order to investigate this effect further, additional needle bore sizes were tested, corresponding to 25G and 30G. These are the types of needle most frequently employed in clinical practice^19^, allowing us to reproduce more faithfully a clinical injection procedure. Overall, decreasing the needle diameter from 16G to 30G resulted in lower cell death (Fig. 3A). In the case of PEM, there was statistically significant difference in foam efficacy between 16G and 30G needles (p = 0.03) (Fig. 3A). Comparing the different foam production methods, statistical difference was found only when using the largest needle (16G), with PEM associated with statistically greater treatment efficacy (% attached cells: 11.8 ± 4.6%) compared to both DSS (% attached cells: 19.5 ± 8.9%) and TSS (% attached cells: 20.0 ± 11.3%) foams.

**Figure 4:**
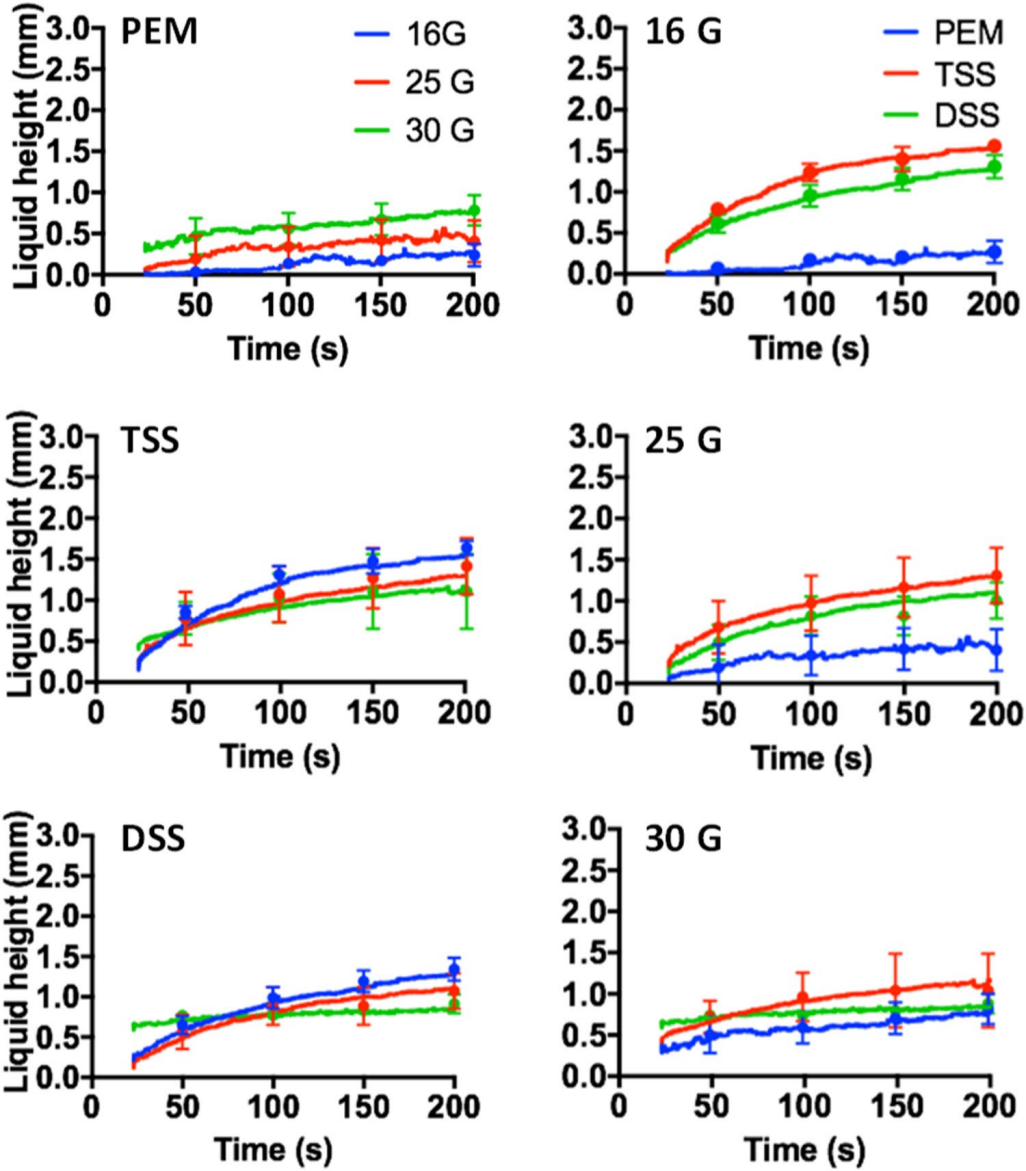
Quantification of the effect of needle bore size on foam drainage dynamics. The height of liquid POL solution at the bottom of the vial was quantified over time (up to 200 seconds; representative time points are shown at 50, 100, 150 and 200 s), using a custom-built Phyton script. On the left column, results are reported to illustrate the comparison between needle diameters for a fixed foam production method [30G (green), 25G (red), and 16G (blue)]. On the right column, results are reported to illustrate the comparison between foam production methods, for a fixed needle diameter [PEM (blue), TSS (red), and DSS (green)]. The experiment was repeated five times, for each condition investigated.

In order to determine the effect of needles on foam physical properties, bubble size measurements were carried out using the glass-plate method. Figure S3 shows the bubble size distribution of PEM and PCF foams, injected through different needle sizes. Results show that injection through a needle did not significantly impact on the bubble size distribution of all types of foam. Comparing the different foam types, room air PCFs had a narrower bubble size distribution than PEM (in the bubble size range 0–400 μm) for all needle inner diameters investigated. However, PCFs had a greater number of bubbles in the size range 400–510 μm compared to PEM. Despite there was no significant change in bubble size distribution, foam injection through a needle caused visible phase separation between the liquid and gaseous phases. Therefore, an experiment was developed to quantify foam drainage dynamics within a vial, upon foam injection through needles of different bore size. The vial inner diameter was comparable to the one of well plates used for *in vitro* biological testing. Figure 4 shows the time evolution of the height of liquid POL solution at the bottom of the vial, which was employed as a metrics for drainage.

When injected using the narrowest needle diameter (30G), all foams presented a higher liquid fraction at the beginning of the experiment [liquid height was 0.45 mm (PEM), 0.66 mm (TSS), and 0.67 mm (DSS)], followed by a relatively slow drainage dynamics. After 200 s, the liquid height was 0.77 mm for PEM, 1.67 mm for TSS, and 0.84 mm for DSS. Differences between foams were more evident at the larger needle diameters, with PEM foam undergoing a significantly slower drainage compared to DSS and TSS foams. The largest difference between foam types was observed when using the 16G needle; after 200 s, the liquid height was equal to 0.23 mm (PEM), 1.52 mm (TSS), and 1.26 mm (DSS).

**Figure 5:**
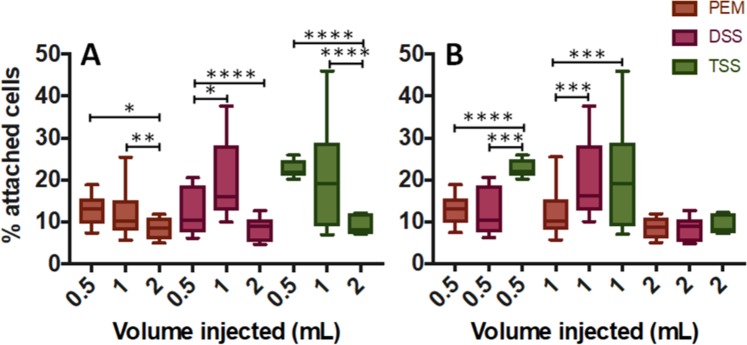
*In vitro* evaluation of the effect of foam volume on HUVECs, using different types of foam. Different foam production methods were investigated, including PEM (brown), DSS (pink), and TSS (green). The volume injected was 0.5 mL, 1 mL, or 2 mL, for each type of foam. Data are reported (Tukey’s box plot) as % of cells attached after treatment (compared to untreated cells), determined via methylene blue method. The effect of injected foam volume (for each foam production method) is illustrated in (**A**), while a comparison between foam production methods (for each foam volume) is illustrated in (**B**). The experiment was repeated four times. One asterisk (*) indicates p ≤ 0.05, two asterisks (**) indicate p ≤ 0.01, three asterisks (***) indicate p ≤ 0.001, and four asterisks (****) indicate p ≤ 0.0001.

The biological effect of changing the foam volume was also investigated, by injecting either 0.5 mL, 1 mL, or 2 mL (which are comparable to clinically injected volumes, if normalised to the treated surface area)^19^. In these experiments, the treatment time was fixed to 15 seconds. Results showed a significant reduction in the percentage of attached cells with increasing the volume of foam from 0.5 mL to 2 mL (Fig. 5). Moreover, PEM had significantly greater efficacy compared to PCFs when using 0.5 and 1 mL of foam. Increasing the foam volume further (2 mL) resulted in comparable percentage of attached cells between PEM and PCFs (<10% in all cases).

**Figure 6:**
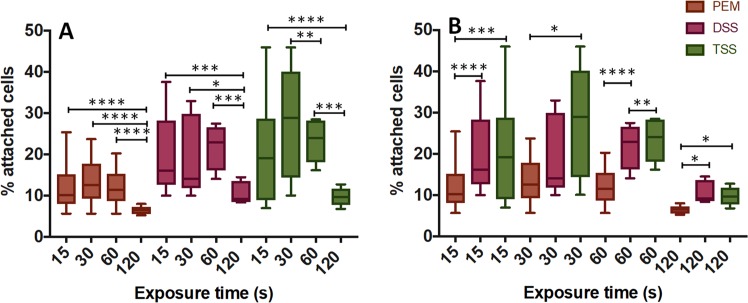
*In vitro* evaluation of the effect of foam exposure time on HUVECs, using different types of foam. Methods of foam production investigated included PEM (brown), DSS (pink), and TSS (green). 1 mL of foam was injected in these experiments, using a 16G needle. Cell monolayers were exposed to each foam for 15, 30, 60 and 120 seconds. Data are reported (Tukey’s box plot) as percentage of attached cells after treatment (compared to untreated cells), determined via methylene blue method. The effect of treatment time (for each foam production method) is illustrated in (**A**), while a comparison between foam production methods (for each treatment time) is illustrated in (**B**). The experiment was repeated ten times. One asterisk (*) indicates p ≤ 0.05, two asterisks (**) indicate p ≤ 0.01, three asterisks (***) indicate p ≤ 0.001, and four asterisks (****) indicate p ≤ 0.0001.

The effect of varying the exposure time of HUVECs monolayers to sclerosing agents was investigated. Earlier *in vitro* and *ex vivo* studies have reported on treatment times in the range 5 s–1 hr, whilst it is usually recognised to be in the order of a few seconds *in vivo*^32^. In this study, the treatment time was varied in the range 15–120 s, which is consistent with our previous determinations of foam plug persistence within an artificial vein model^17^. As shown in Fig. 6, the efficacy of a 120 s long treatment (PEM = 6.5 ± 0.9%, DSS = 10.5 ± 2.6%, TSS = 9.7 ± 2.3%) was significantly higher compared to shorter treatments. Overall, PEM was statistically more effective than both DSS and TSS, at all treatment times investigated.

**Figure 7:**
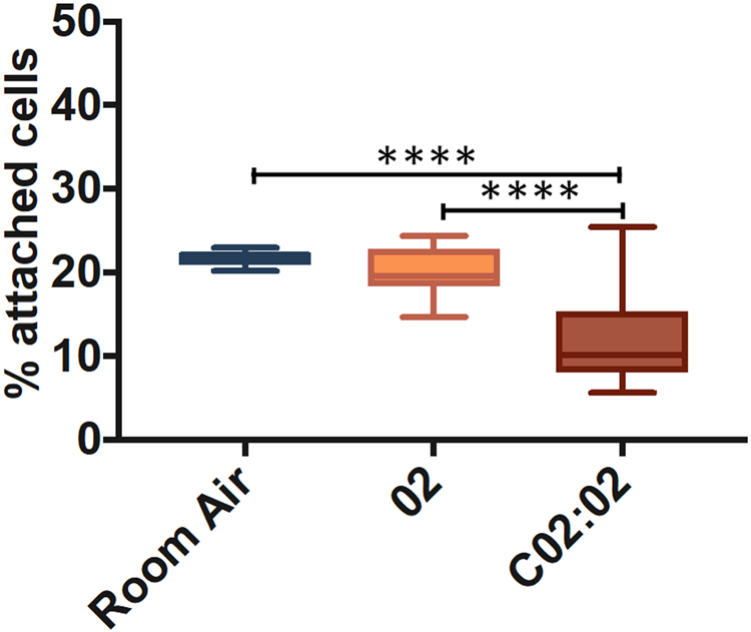
*In vitro* evaluation of the effect of PEM gas formulation on HUVECs. 1 mL of PEM foam was injected in these experiments, using a 16 G needle. Cell monolayers were exposed to each foam type for 15 seconds. Foams tested were PEM containing either room air, 100% O_2_, and 35:65 CO_2_:O_2_. Data are reported (Tukey’s box plot) as percentage of attached cells after treatment (compared to untreated cells), determined via methylene blue method. The experiment was repeated twenty times. Four asterisks (****) indicate p ≤ 0.0001.

In a final series of experiments, the effect of the gas formulation was investigated by comparing the efficacy of PEM foams containing either 35:65 CO_2_:O_2_ (conventional PEM formulation), RA, and 100% O_2_. The 35:65 CO_2_:O_2_ PEM had significantly greater efficacy (11.8 ± 4.6% of cells attached) compared to RA (21.8 ± 0.9%) and 100% O_2_ (20.5 ± 2.9%) PEM formulations (Fig. 7).

**Figure 8:**
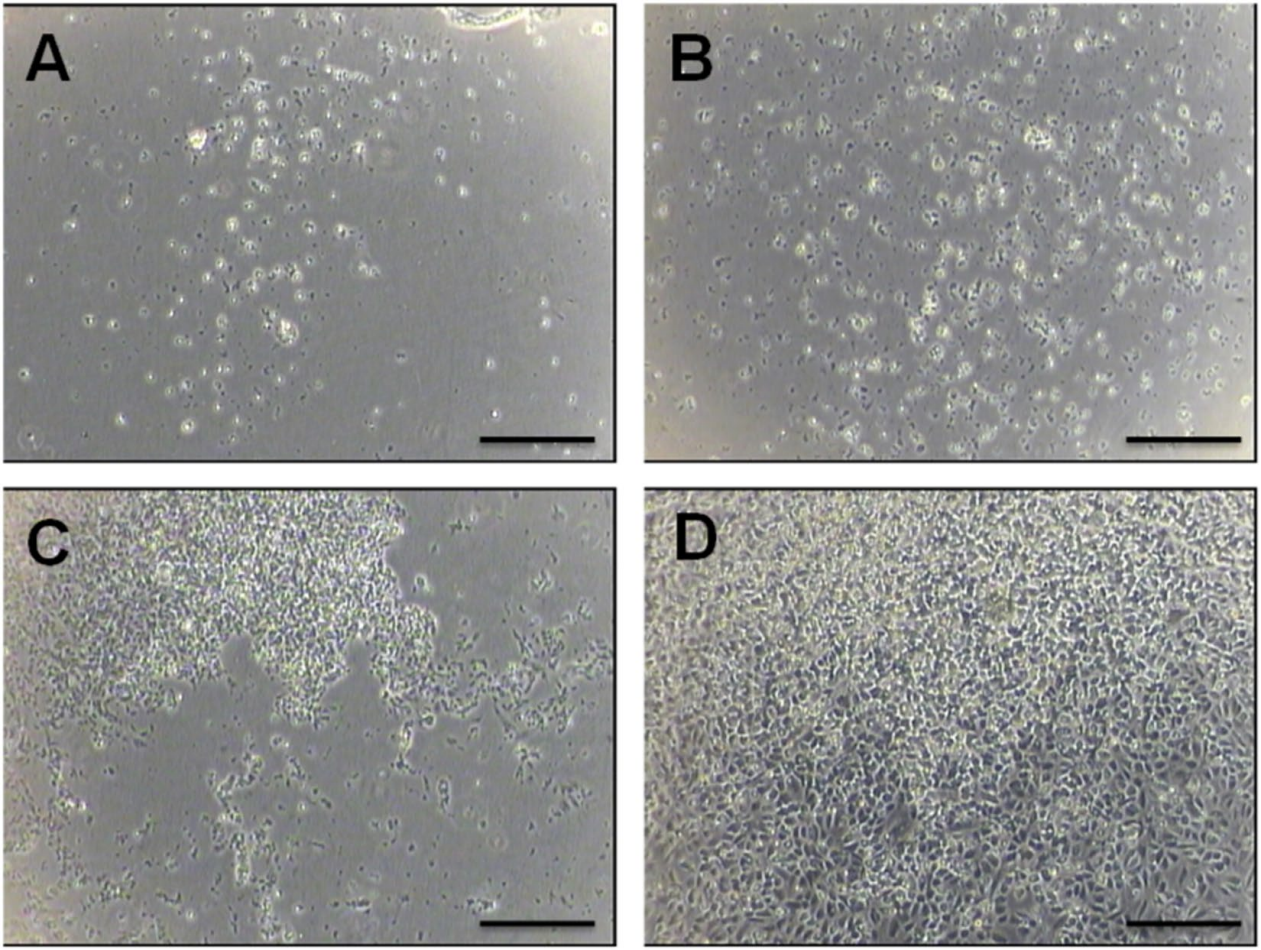
Histopathologic observation of HUVECs upon treatment with sclerosing foams. Microscope images (4x magnification) illustrate HUVECs monolayers treated for 15 seconds using PEM (**A**), DSS (**B**), Tessari (**C**) foams, and untreated (**D**). Scale bars are 200 µm.

In addition to the above quantitative assays, histopathologic observations of treated HUVECs were performed. Images of cell monolayers exposed to various sclerosing agents were captured, using an optical microscope with phase contrast. The untreated (control) cells displayed a normal EC morphology for confluent monolayers, and were adherent to the substrate (Fig. 8(D)). Following treatment, cell morphology changed to a more rounded appearance; the monolayer became disrupted, where a large number of cells detached from the substrate and, in some cases, only fragments of cells were present. Figure 8(A–C) show images of cells after exposure to foam generated using different production methods (15 seconds treatment duration, and 1 mL of foam injected without needle). It is evident that PEM (Fig. 8(A)) and DSS RA (Fig. 8(B)) foams caused greater cell detachment compared to TSS foam (Fig. 8(C)), which is coherent with the quantitative determinations (Fig. S1B).

### *Ex vivo* evaluation of the biological performance of sclerosing agents

**Figure 9:**
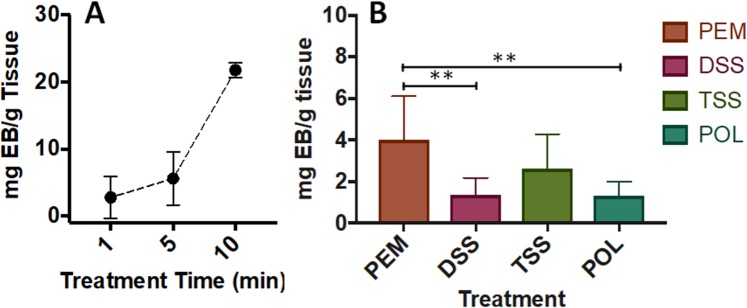
*Ex vivo* evaluation of the effect of liquid and foamed polidocanol on umbilical cord veins. (**A**) Evaluation of the effect of treatment time on umbilical cord vein, using liquid polidocanol (2 mL, for 2 cm vein segment). The vein wall was exposed to polidocanol for 1, 5 and 10 minutes. Data are reported as mg of EB per grams of tissue, determined via Evans Blue method. The experiment was repeated four times. (**B**) Evaluation of the effect of foam on umbilical cord vein, using different types of sclerosing agent: PEM, DSS, TSS, and liquid POL (2 mL, for 2 cm vein segment). The vein wall was exposed to the sclerosing agents for 1 minute. Data are reported as mg of EB per grams of tissue, determined via Evans Blue method. Two asterisks (**) indicate p ≤ 0.01.

In order to investigate the sclerosing performance of foams in a more realistic biological model, *ex vivo* experiments were established. The reliability of the method was initially evaluated by quantifying endothelial damage induced by Type I collagenase, an enzyme that removes EC from the vessel wall by proteolysis of underlying collagen. The vein was exposed to the enzyme for 10 minutes. The same procedure was repeated using liquid POL (1% v/v), and a physiological saline as a control (Fig. S4). Following exposure to Evans Blue-conjugated BSA, control cords showed no leakage into the tissue surrounding the vein (the quantity of EB extravasated was 0.5 ± 0.2 mg EB/g tissue). The collagenase solution (positive control) showed a level of disruption equivalent to 42 ± 4.5 mg EB/g tissue, whereas liquid POL caused 21 ± 1.2 mg EB/g tissue of extravasation. Upon verification of the method, the effect of treatment time was investigated. The vein was treated with liquid POL 1% for 1, 5, and 10 minutes. Figure 9(A) shows that endothelial disruption is directly proportional to exposure time (extravasation ranged from 1.55 ± 2 to 21 ± 1.2 to mg EB/g tissue).

The same experiment was subsequently performed using PCFs and PEM, using a constant exposure time of 1 minute. Figure 9(B) shows that PEM was more effective in disrupting the endothelium compared to DSS PCF and liquid POL; whilst no significant difference was observed between PCFs and liquid POL. The DSS method in this test produced less disruption than PEM (corresponding to 1.3 ± 0.8 and 3.9 ± 2.1 mg EB/g tissue, respectively) but the variation in the measurement was such that this was not statistically significant.

## Discussion

### Effect of foam production methods and administration-related parameters on foam efficacy *in vitro*

Since the introduction of foam sclerotherapy as a treatment method against varicose veins, numerous studies have been conducted in order to further the understanding of the physical properties and behavior of foams^20,23,33,34^. However, a relatively limited body of work has focused on the biological effects of sclerosants on endothelial cells and the vessel wall^7,11,25,26^. Earlier studies have revealed that sclerosing efficacy is directly correlated to treatment time and sclerosant concentration^29,35^. However, the lack of quantitative analyses and the difference between the physical properties of sclerosing agents investigated, have both hindered the ability to draw generalized conclusions about the efficacy of different foam production and administration methods.

In this study, we have employed two simple quantitative methods to compare the microscopic and macroscopic effects of different foam production techniques on the endothelium. With respect to the microscopic effects, we proposed an *in vitro* model that allows the quantification of sclerosant-induced endothelial disruption, by determining the number of cells attached to a substrate after treatment. In this method, monolayers of endothelial cells provide a simplified replica of a small segment of vascular endothelium. The experimental protocol has been designed to mimic the different treatment phases occurring *in vivo*, i.e. (i) Injection of the foam and its contact with the endothelium, and (ii) washing out of the foam due to blood flow. Being a biological model within a static fluidic environment, foam-induced blood displacement occurring *in vivo* is reproduced by an active washing phase. With this model, clinically relevant procedural parameters have been investigated, such as volume of foam injected, treatment time, and usage of different types of needle.

The repeatability of the method was initially evaluated, showing significant consistency across multiple independent repeats (Fig. S1A). In a first step of the study, the model was utilised to investigate the sclerosing efficacy of liquid POL. Only at volumetric concentrations <0.02% the surfactant was rendered ineffective, confirming the potency of this detergent at disrupting the endothelial cell membrane and inducing cell death^27^ (Fig. 1). Serial treatments using the same POL solution were performed to assess whether polidocanol deactivation occurred. Reducing the number of active molecules (i.e., by lowering the POL concentration) caused reduced efficacy after a certain number of treatments, which was dependent upon the POL concentration (see Fig. 2). Depletion of active polidocanol over consecutive treatments was likely due to its intercalation within lysed membrane fragments. However, the 1% POL solution (employed to manufacture both PCF and PEM foams) maintained its potency across multiple treatments, and its *in vitro* biological performance was not affected by polidocanol depletion.

Upon verification of polidocanol efficacy *in vitro*, the effect of administering sclerosing foams with needles of different bore diameter was investigated. The needle bore size is typically selected based on the vein to be treated, with smaller veins often requiring the smaller 25–30G needles^19^. The performance of different sclerosing foams was statistically different only when using the larger needle (16G) (Fig. 3). Employing narrower needles (i.e., 25G and 30G), foam efficacy reduced and differences between foam types were not statistically significant. This observation may be due to changes in the physical properties of foams when they were conveyed through a needle. Bubble size measurements however revealed that the bubble size distribution of all types of foam was virtually unaffected by the needle inner diameter (Fig. S3). Previous studies have shown that as foam flows through a pipe, the change in bubble diameter is dependent on the pressure drop across the pipe^36^. It can therefore be inferred that the pressure drop required to administer foams manually through clinical needles – and the resultant shear rate – were not sufficient to cause a significant change in the bubble size of PEM and PCF foams. Thus, the observed changes in foam therapeutic efficacy could not be directly related to the foam bubble size distribution. For this reason, additional experiments were performed to quantify the effect of needle injection on foam drainage dynamics, where drainage describes the flow of liquid through a foam^37^. During free drainage, the liquid volume fraction increases monotonically from the top to the bottom of a foam column. This bottom liquid layer is depleted of surfactant molecules, as the surfactant preferentially stabilises the gas-liquid interface of bubbles located in the upper foam layer. The liquid then continues to drain downward over time, until the liquid height reaches a steady state^38^ (as shown in Fig. 4). Given that drainage is strongly affected by the size and shape of the foam container, a vial with inner diameter comparable to the well plate used in biological tests was employed. By injecting foams through the narrowest needle (30G) caused visible separation of the liquid and gaseous phases upon injection; thus, the ejected foam experienced only limited drainage (Fig. 4). Phase separation may occur because of the liquid POL travelling at a different velocity compared to the gas bubbles, as observed for other multi-phase systems delivered through needles, such as pastes and cements^39^. The extent of phase separation reduced with increasing the needle inner diameter (corresponding to lower injection velocity), and was almost absent when foams were administered using the largest 16G needle (Fig. 4). When foam separation occurred (i.e., using the 25G and 30G needles), the biological efficacy of foams was dominated by their ‘static’ liquid fraction, and differences between foam types were not statistically significant (Fig. 3). Conversely, when phase separation was significantly reduced (as in the 16G needle experiments), the ejected foams displayed distinct drainage dynamics (see Fig. 4) that in turn led to differences in their biological efficacy. Notably, the slower drainage of PEM foam resulted in statistically greater therapeutic efficacy compared to PCF foams (Fig. 3), which instead presented a faster initial drainage dynamics. The more rapid drainage of room air PCF foams could be attributed to: (i) the greater liquid:gas volume ratio compared to PEM foam^22^, with previous studies reporting on a direct correlation between foam drainage velocity and liquid fraction^40^. (ii) The lower average bubble diameter combined with the presence of a greater proportion of bubbles with diameter >400 μm (see Fig. S3). Notably, higher pressure within the smaller bubbles drives diffusive gas exchange towards the larger bubbles, and the resulting coarsening of the foam accelerates its initial drainage dynamics^41^.

The mechanism for which the slower foam drainage of PEM leads to greater therapeutic efficacy *in vitro*, is not fully understood yet. However, it could be attributed to the persistence of gas bubbles in the vicinity of the cell membrane, with higher concentration of active polidocanol located at the gas-liquid interface. Conversely, when a fast-draining foam is employed, cells are exposed to the liquid phase that has been depleted of polidocanol, particularly in the shorter term. Depletion is greater in N_2_-containing foams, given to the lower ‘mobility’ of surfactant molecules in these foams^42^.

The effect of the injected foam volume was also investigated, as it represents a parameter that is varied in the clinical practice. Generally, the volume injected is dependent on the diameter and length of the vein to be treated^43^. There was a significant difference between foam production methods when injecting 0.5 and 1 mL of foam, whereas all treatments had very similar biological performance and became more effective when injecting a greater volume of foam (2 mL) (see Fig. 5). Earlier studies have reported that the dependence of drainage time on the foam liquid fraction reduces with increasing the height of a foam column^44^, which may explain the comparable efficacy of PEM (liquid fraction: 12.5%) and PCFs (liquid fraction: 20%) at 2 mL. The positive correlation between the injected volume and treatment efficacy may be attributed to increased gravitational effects at the higher foam heights^40^, which favors downward motion of active polidocanol towards the cell monolayer. It should be noted that a foam volume ≤1 mL is more representative of a clinical injection procedure, considering the volume of foam normalised to the area of the treated endothelial layer^19^. At these lower volumes, drainage dynamics is governed by both capillarity and gravitational effects.

The effect of varying the treatment time was also investigated. The exposure time was defined based on the predicted persistence of a foam plug *in vivo*^17^, and values investigated were 15, 30, 60 and 120 seconds (Fig. 6). Overall, there was significant difference in biological efficacy between 15, 30, and 60 seconds of exposure. However, for all types of foam, efficacy significantly increased at 120 seconds of exposure. Notably, bubble collapse in the longer term causes a release of active polidocanol, and biological effects thus become dependent on the liquid POL solution. Further investigations are required to fully elucidate the interplay between foam drainage and the temporal dynamics of membrane disruption upon exposure to the surfactant agent. Overall, PEM maintained superior performance across the all range of exposure times investigated and was more effective over longer term exposures, likely due to its sustained drainage dynamics compared to PCFs^17^.

Considering the potency of the 1% POL solution over multiple treatments *in vitro* (as illustrated in Fig. 2), the greater therapeutic efficacy of PEM compared to PCFs may not be solely attributed to differences in foam stability and drainage dynamics. Previous studies have demonstrated that the diffusion velocity (or mobility) of water-soluble surfactants in foams is affected by the gas formulation, and that it is greatest in CO_2_ foams, followed by O_2_ foams and N_2_ foams^42^. Experiments were thus conducted using PEM manufactured using different gas formulations (Fig. 7), to assess whether changes in surfactant mobility may influence its therapeutic efficacy. Coherently with these previous findings, the commercial PEM formulation (35:65 CO_2_:O_2_) had greater efficacy than both 100% O_2_ PEM and N_2_-containing (RA) PEM. These results suggest that polidocanol is more readily available for interaction with cell membranes, when N_2_-free foams are employed.

### Comparing the *ex vivo* performance of different foam production methods

In order to evaluate the performance of different sclerosing agents in a more realistic biological model, experiments were performed *ex vivo* using umbilical cord veins. Sclerosant-induced disruption of the endothelium was determined from extravasation of a BSA-conjugated dye. Initially, the ability of the method to provide a quantification of endothelial disruption was assessed, using collagenase to actively cause endothelial damage. Collagenase, an endopeptidase that digests native collagen^45^, was left in the vein for 10 minutes (Fig. S4). The same procedure was performed using liquid POL (1% v/v) or physiological saline as a control. As expected, saline did not cause tissue damage, whereas the collagenase solution caused greater endothelial disruption compared to liquid POL. It is well known that the enzyme cleaves collagen bonds causing a removal of the endothelium and potential damage to the underlying tissues, compared to a surfactant agent that interferes with the cell membrane only, causing cell death^27^. After method’s validation, more clinically relevant exposure times were applied. Veins were treated with liquid POL 1% for 1, 5 and 10 minutes. A direct correlation between contact time and endothelial disruption was observed (Fig. 9A), consistently with *in vitro* experiments using sclerosing foams.

The same procedure was performed using PCFs and PEM, with an exposure time of 1 minute. PEM was more effective at disrupting the endothelium compared to DSS PCF, as expected from the results obtained *in vitro*. There was also a significant difference between the efficacy of foamed and liquid POL, suggesting that the dynamics of foam drainage and the ‘local’ surfactant concentration levels may become even more influential over foam therapeutic efficacy within a 3D environment. Interestingly, despite TSS foam being less effective in generating endothelial wall damage compared to PEM, differences between mean values were not statistically significant. This finding is in contrast with the *in vitro* results, where DSS foam was consistently superior to TSS, although differences between PCFs significantly reduced with increasing the treatment time both *ex vivo* (Fig. 9B) and *in vitro* (Fig. 6).

### Conclusive remarks

To the best of the authors’ knowledge, the present study represents the first systematic comparison of the biological performance of different sclerosing foam formulations, and a first attempt to correlate biological performance with foam physical properties.

Overall, analyzing the results obtained using both the *in vitro* and *ex vivo* models, PEM was the most effective foam for disrupting the endothelial layer in a variety of tests and over different timescales of treatment. This was attributed to the slower drainage dynamics of PEM compared to PCFs, and – potentially – to the enhanced polidocanol mobility conferred by its gas formulation. It was also shown that reducing the injection needle diameter, increasing the volume of injected foam, and increasing the treatment time, all contributed towards increasing treatment efficacy (for all types of foam).

It should also be highlighted that PCFs made from room air have associated risks, with persistent nitrogen bubbles in the circulation, whereas PEM, made with a low-nitrogen CO_2_:O_2_ gas mixture, is not associated with the risks of high-nitrogen content^46^.

In conclusion, in this study we have developed a simple 2D *in vitro* method to quantify the efficacy of foam sclerotherapy. The method allows for the investigation of different clinical parameters such as exposure time, injected volume, concentration of sclerosant, and needle bore size amongst others. In addition, we utilized a more realistic biological model, i.e. a three-dimensional *ex vivo* vein model, as a further method of evaluation. However, we are aware that the both *in vitro* and *ex vivo* models do not fully reflect the clinical setting for foam sclerotherapy, because they are employed in static conditions and thus do not replicate foam-induced blood displacement, and also do not provide a faithful replication of the varicose vein architecture. Our group is therefore working to overcome this limitation by developing a 3D *in vitro* platform applied in dynamic conditions, moving closer to models that mimic the physiological and clinical environments, ultimately, as an alternative to animal testing. Despite the current limitations, the two models generated reliable and reproducible results, and they can be employed in parallel in order to compare the performance of sclerosing treatments. In our series of comparisons using both models, we confirmed findings from our previous physical studies^17,22,47^ that PEM presents a better overall performance compared to PCFs across a variety of biological efficacy tests.

## Methods

### Foam production methods

In this study, the commercially available Varithena 1% varicose vein treatment (referred to as polidocanol injectable foam or PEM) was employed, and its performance compared with physician compounded foams (PCFs) made using different foam generation methods.

With respect to PCFs, POL (Croda, Goole, UK) at a concentration of 1% (v/v in buffered saline) was employed as a surfactant agent. Foams were produced by mixing liquid and room air (at a volume ratio of 1:4, respectively) as this is the most widely used formulation adopted in clinical practice^48^. Two methods of PCF production were investigated: (i) DSS and (ii) Tessari. In the DSS method, foam was produced by passing the POL solution from a 5 mL syringe, ten times into and out of a 10 mL syringe. Silicon-free syringes (BD Biosciences, USA) were connected *via* a Combidyn adapter (B. Braun Melsungen, Germany). In the Tessari method, the straight connector was replaced with a three-way valve that was set at a 30° off-set. Polidocanol endovenous microfoam (PEM) Varithena is a commercially available microfoam combination produced by Provensis Ltd (a BTG International group company, London, UK) consisting of a proprietary 35:65 CO_2_:O_2_ gas mixture with ultralow nitrogen content (<0.8%) and 1% POL solution. The foam is contained within a pressurized canister combined with a transfer unit, which can be connected to a 10 mL silicone-free syringe. Once connected, the syringe is filled with 5 mL of foam. Experiments were conducted at room temperature (23 °C), after foam production, and foams were produced by the same operator.

### *In vitro* test method to evaluate performance of sclerosants

A method was designed to test the efficacy of sclerosants, in both their liquid and foamed form. A monolayer of human umbilical vein endothelial cells (HUVECs) was cultured until confluence into 24 well-plates (Sigma-Aldrich Co. LLC., USA). The following steps were designed to mimic different phases of sclerosant’s injection: (i) the HUVECs media (HM) (Thermo Fisher Scientific Inc., USA) was removed from the wells, in order to achieve direct contact between cells and sclerosants; (ii) the cell monolayer was exposed to various sclerosing agents during a fixed time of approximately 15 seconds, reproducing the injection process; (iii) sclerosants were removed using a pipette, and cells were washed once using a HBSS buffer (Hanks Buffered Saline Solution, Sigma-Aldrich Co. LLC., USA) mimicking the sclerosant’s displacement and dilution caused by blood flow; and (iv) fresh medium was added. Sclerosants’ injection was performed manually using a 5 mL syringe (BD Biosciences, USA), with and without a needle. The syringe was kept perpendicular to the bottom plane of the well, and the sclerosing agent was injected from the centre of the well. The standard procedure was carried out under these conditions: 1 mL of liquid/foamed sclerosant, 15 seconds of exposure time, and a 16 G needle employed for injection. Following treatment, the medium was removed and cells were washed gently in warm HBSS, which was subsequently removed by aspiration.

Cells were subsequently fixed with the addition of 0.7 mL of a 10% formyl saline solution (Sigma-Aldrich Co. LLC., USA). Fixative was then removed by aspiration, and a methylene blue solution (MB) 1% (w/v methylene blue in 0.01 M-borate buffer pH8.5) (Sigma-Aldrich Co. LLC., USA) was added to each well.

The MB solution was then transferred to a 96 well flat-bottomed plate (Sigma-Aldrich Co. LLC., USA), with 0.1 mL being added in duplicate wells. A control set of untreated cells was used to generate a standard curve of MB equivalent to serial dilutions of 100% cells. MB absorbance was then measured using a plate reading spectrophotometer, at a wavelength of 650 nm. Absorbance values of treated cells were then converted into a percentage of attached cells, using a calibration function. The number of cells attached is a measure of the number of live cells upon treatment. The latter was derived from linear regression of experimental data points, using Prism software (GraphPad Software, Inc., USA).

During the study different parameters were varied, such as (i) volume injected, (ii) exposure time, (iii) needle bore size, and (iv) gas formulation. The volumes of injected sclerosant investigated were 0.5, 1, and 2 mL, whilst the exposure times investigated were 15, 30, 60 and 120 seconds. The needles employed were selected based on the common clinical practice, and had an inner diameter of 30G, 25G and 21G, corresponding to 0.16, 0.26 and 0.51 mm, respectively (BD Biosciences, USA). In order to investigate the effect of the gas formulation, PEM foam was produced using different gas constituents (in addition to the commercial formulation), including 100% O_2_ and room air.

### Measurement of foam drainage dynamics

A transparent glass vial (outer diameter: 10.9 mm) was placed within a custom-built photographic chamber with a black background. A charge-coupled device (CCD) camera (Canon EOS06) was positioned in front of the vial. The foam was produced and injected (2 mL) inside the vial, using different types of needle (16G, 25G, and 30G) and different foam production techniques (PCFs and PEM). The experiment was repeated five times, for each condition investigated.

The time between foam injection and the beginning of the video recording was approximately 23 seconds. Videos were recorded for 5 minutes (25 frames per second), and subsequently analysed using a Phyton script developed in-house. The script loads the video and extracts its individual frames. It then performs the following steps in a semi-automated fashion:

(i) User selection of a region of interest for analysis.
(ii) Calibrating the image dimensions, by converting pixels into physical units. This is carried out by user selection of a feature of known length (for instance, the diameter of the vial).
(iii) Converting the image into a black and white binary format, where black corresponds to the liquid phase and white corresponds to foam.
(iv) The centerline of the selected region of interest is determined, and a rectangular window for analysis is defined. The width of this window extends 5 pixels away from the centerline, at both sides. It was decided to analyse foam drainage within an interrogation window (as opposed to a line), as data would be less sensitive to experimental noise.
(v) Automated counting of the number of black pixels along the height of the interrogation window. An average height was determined, which corresponded to the height of liquid POL in the vial (upon dimensional calibration).
(vi) Steps (iii)–(v) were performed automatically on each image frame, and a plot of the liquid height (in mm) *vs*. time was generated. This provided a quantitative measure of foam drainage dynamics.

### Measurement of bubble size distribution

The bubble size distribution was measured using an in-house glass-plate method, as described in our earlier study^22^. Briefly, an aliquot of freshly generated foam (volume: 49 *μ*L) was placed on a glass plate and immediately covered by another. The plates were thick enough not to bend, and were separated by a 32 *μ*m thick gap.

A flattened foam monolayer was thus created, which comprised 32 *μ*m high, flat cylindrical bubbles. A light microscope and camera (AxioCam ICc 1, Carl Zeiss Microscopy, Cambridge, UK), with lighting adjusted to create sharp images of circular boundaries, were employed to capture sequential image fields. A built-in software was used to “stitch” fields together. Each individual bubble was identified and the bubble diameter measured using the image analysis (AxioVision, Zeiss) programme, with bespoke BubbleSizerMeasure macro. Approximately 2000–3000 bubbles per sample were measured using this procedure. The experiment was repeated five times, for each condition investigated.

### Microscope imaging of treated cells

Bright field images of HUVECs were acquired with an optical microscope (Olympus, CKX41, Japan). Images were taken of live samples immediately after treatment, with phase contrast microscopy (objective magnification 4x).

### *Ex vivo* test method to evaluate performance of sclerosants

This part of the study was carried out in accordance with the Human Tissue Act (2004) and the recommendations of Southampton & South West Hampshire Research Ethics Committee B with Governance provided by the University of Southampton Research Governance Office. Umbilical cords were collected from the Princess Anne Hospital (Southampton, UK) from non-complicated natural vaginal births following agreed ethical collection protocols (Local Research Ethical Committee (LREC); Ref: 07/H0502/83). The umbilical cord was cut from the placenta and sectioned into 10 cm long segments. A steel feeding cannula (16G) was inserted into the vein. The cannula was clamped in place and attached to a 30 mL syringe filled with a physiological saline “cord buffer”. The vein was washed until the fluid exiting the other end of the cord was clear. The treated umbilical sample was then cut into 5 vein segments. The vein was filled with a collagenase solution at 0.1% in phosphate buffered saline (PBS, Worthington Biochemical Corp., USA) or with different types of sclerosing agent. The cord segment was then incubated at 37 °C for 10 min. After incubation, the vein was washed again with cord buffer, and filled with 2 mL of Evans blue (EB) (0.33% EB and bovine serum albumin, BSA). The cord was then incubated at 37 °C for 20 min. After incubation, Evans blue was washed out using the cord buffer.

Each cord segment was cut in smaller pieces (0.5 cm long), which were weighed and inserted in 1.5 mL tubes. A formamide solution was added into each tube, and all tubes were transferred into a 62 °C water bath and incubated overnight in order to extract EB from the tissue. Tubes were centrifuged at 13000 rpm for 20 minutes at 20 °C. The supernatant was then transferred into a 96 well flat-bottomed plate. The EB stock solution was serially diluted to generate a standard curve. EB absorbance was then measured on a plate reading spectrophotometer, at a wavelength of 610 nm. The absorbance from a calibration standard curve was used to calculate unknowns, using the Prism software (GraphPad Software, Inc., USA) and applying a hyperbolic interpolation and regression.

Afterwards, the amount of extravasated Evans blue (in mg) per gram of tissue was calculated.

### Statistical analysis

The comparisons between treatments were performed using unpaired Student’s t-test with Welch’s correction, with appropriate post-hoc tests. Statistical significance was assumed for p < 0.05. All statistical tests were performed with Prism software. Data were reported either as the mean ± standard deviation, or in the form of a Tukey’s box plot (comprising 25^th^ percentile, median, and 75^th^ percentile).

## Supplementary information

Supplementary Information
