# Supplementary material for: In vitro and ex vivo evaluation of the biological performance of sclerosing foams
Source: Sci Rep. 2019 Jul 8;9:9880. doi: 10.1038/s41598-019-46262-0 (PMC6614483; doi:10.1038/s41598-019-46262-0)
Supplement: Supplementary file 1 — Supplementary Information [file 41598_2019_46262_MOESM1_ESM.pdf]

Supplementary Information for:

In vitro and *ex vivo* evaluation of the biological performance of sclerosing foams.

Elisabetta Bottaro[1], Jemma A. J. Paterson[2], Luciano Quercia[3], Xunli Zhang[1,4], Martyn Hill[1,4], Venisha A. Patel[5], Stephen A. Jones[5], Andrew L. Lewis[5], Timothy M. Millar[2*], Dario Carugo[1,4*]

[*] Corresponding authors

[1] Faculty of Engineering and Physical Sciences, University of Southampton, Southampton, UK

[2] Faculty of Medicine, University of Southampton, Southampton, UK

[3] Computer Science Department, University of Bari, Italy.

[4] Institue for Life Sciences (IfLS), University of Southampton, Southampton

[5] Biocompatibles UK Ltd., Lakeview, Riverside Way, Watchmoor Park, Camberley, UK

* Corresponding authors: Timothy M. Millar (T.M.Millar@soton.ac.uk) and Dario Carugo (D.Carugo@soton.ac.uk)

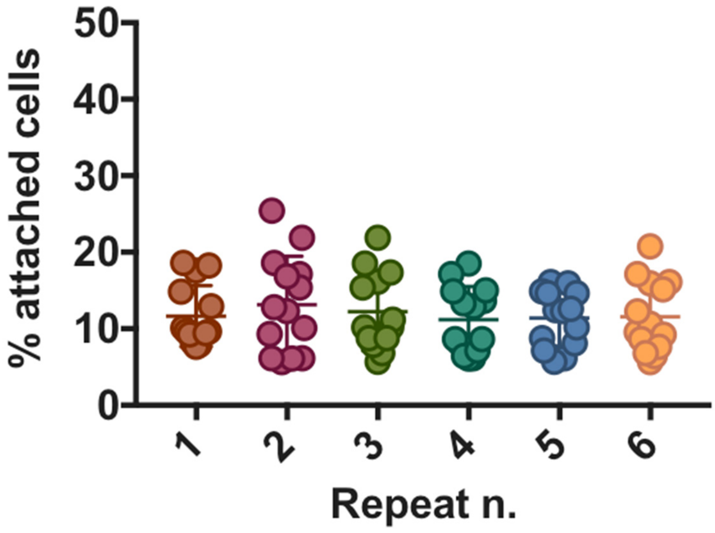

Figure S1. ***Evaluation of the reproducibility of the in vitro test method.*** *1 mL of PEM was injected onto the ECs monolayer, using a 16G needle. The exposure time to the sclerosing foam was set to 15 s. The experiment was repeated six times.*

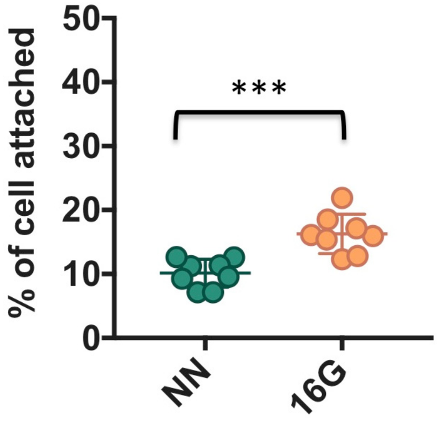

Figure S2. ***In vitro evaluation of the effect of a needle during foam injection.*** *PEM (15 s exposure time, 1 mL) was injected without (NN, filled circles) and with a 16 G needle (filled squares). Data are reported as % of attached cells (compared to untreated cells) after treatment, determined via methylene blue method. The experiment was repeated eight times. Three asterisks (***) indicate that $p < 0.001$.*

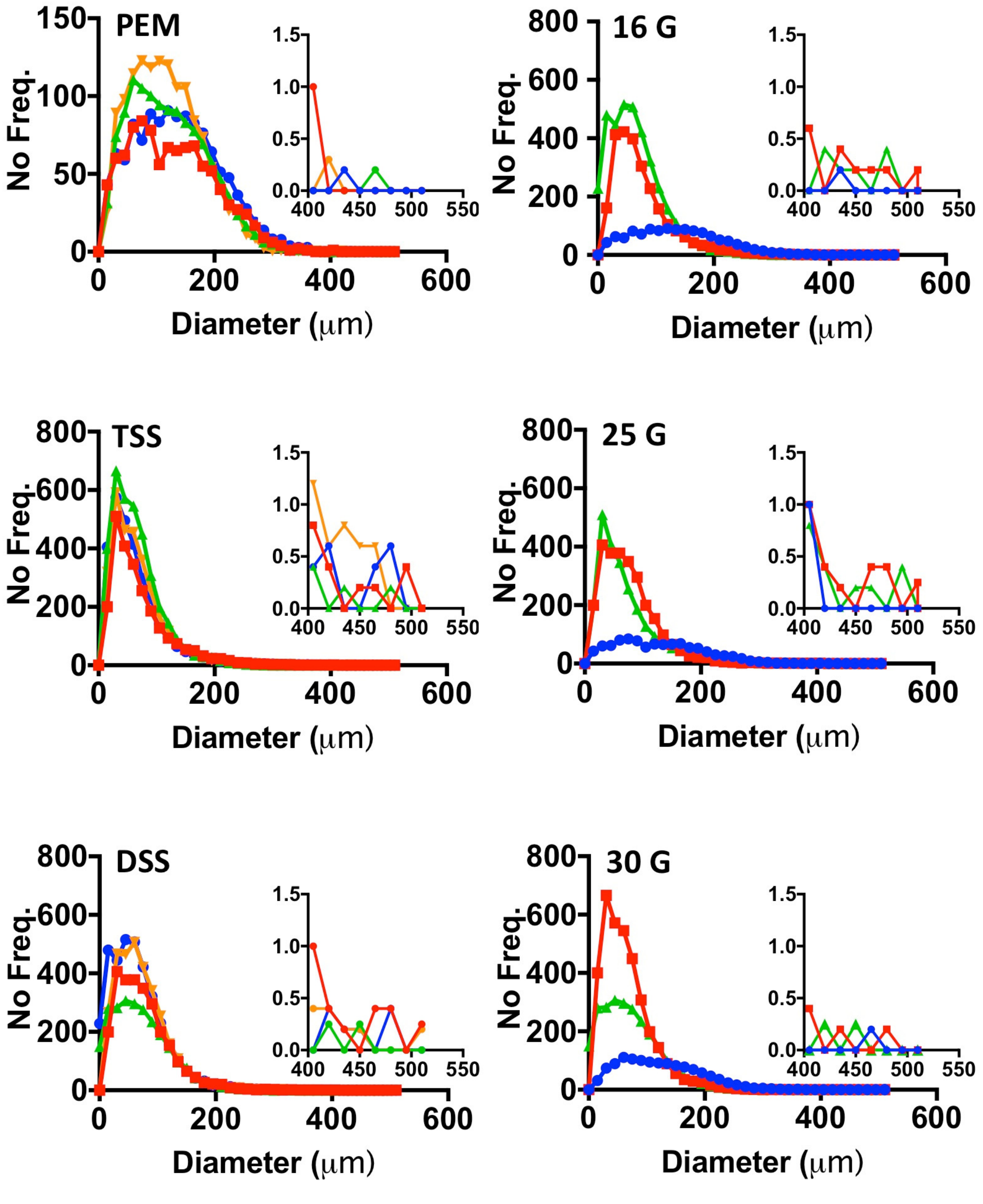

Figure S3. ***Quantification of the effect of needle bore size on bubble size distribution, for both PCFs and PEM.*** *Bubble size distribution was measured using the glass-plate method, and is reported in the form of a frequency plot. The comparison between different needle inner diameters, for a fixed foam production method, is reported on the left column [30G (green), 25G (red), 16G (blue), no needle (orange)]. The comparison between different foam production methods, for a fixed needle inner diameter, is instead reported on the right column [PEM (blue), TSS (red), and DSS (green)]. Each experimental condition was repeated five times.*

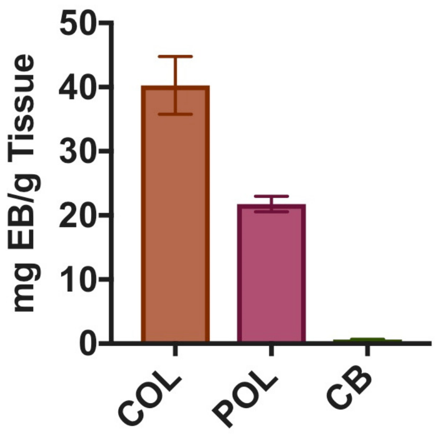

Figure S4. ***Ex vivo validation of the Evans Blue (EB) method.*** *Evaluation of the effect of collagenase, liquid polidocanol, and cord buffer as a control (2 mL, for 2 cm vein segment). The vein wall was exposed to different sclerosing agents for 1 minute. Data are reported as mg of extravasated EB per grams of tissue.*
